# Antimicrobial Photodynamic Therapy in Diabetic Amputation Ulcers: Case Series Evaluating Wound Healing Quality

**DOI:** 10.1002/lsm.70037

**Published:** 2025-06-22

**Authors:** Priscilla Farias Chagas, Thais Barbosa dos Santos, Gesiane dos Santos Trivino, Sandra Kalil Bussadori, Cinthya Cosme Gutierrez Duran, Kristianne Porta Santos Fernandes, Rebeca Boltes Cecatto, Anna Carolina Ratto Tempestini Horliana, Raquel Agnelli Mesquita‐Ferrari

**Affiliations:** ^1^ Postgraduate Program in Biophotonics ‐ Medicine Universidade Nove de Julho, UNINOVE São Paulo São Paulo Brazil; ^2^ Primary Care Coordination (CAP) Program Area 5.1 of Rio de Janeiro Rio de Janeiro Brazil; ^3^ Postgraduate Program in Rehabilitation Sciences Universidade Nove de Julho, UNINOVE São Paulo São Paulo Brazil

**Keywords:** antimicrobial photodynamic therapy, Bates‐Jensen, contamination, diabetic foot, infection, wound

## Abstract

**Introduction:**

Diabetic foot ulcers affect approximately 15% of individuals with diabetes, compromising their quality of life and placing an additional burden on the public health system.

**Objectives:**

This study aims to evaluate the effects of the antimicrobial photodynamic therapy (aPDT) in tissue repair through the analysis of two case studies involving individuals with diabetes‐related foot lesions.

**Methods:**

aPDT was performed using 1% methylene blue, a cluster of four red lasers (660 nm) with an average radiant power of 100 mW, and radiant energy per emitter of 6 J. The exposure time was 1 min, resulting in a total radiant energy per session of 24 J. Patients were evaluated on sessions 1, 3, 5, 10, and 30 days after the completion of treatment.

**Results:**

Using the Bates‐Jensen Wound Assessment Tool (BWAT) scale as a tool to assess wound progression, Patient 1 had an initial score of 37 points, which reduced to 24 points by the end of treatment, resulting in a decrease of 13 points on the scale. Patient 2 started with 36 points and finished with 14 points, achieving complete healing.

**Conclusions:**

Treatment led to improved wound quality, evidenced by a reduction in BWAT scores, a 74.9% decrease in wound area in patient 1 with consistent healing progress, and complete wound closure in patient 2. Although limited by its case‐series design, this study contributes preliminary clinical evidence suggesting the potential benefits of antimicrobial photodynamic therapy (aPDT) in the management of diabetic foot ulcers and highlights the need for larger, controlled trials to validate its efficacy. In conclusion, aPDT was effective across all evaluated outcomes in these two reported cases.

**Trial Registration:**

NCT06416462 (initial release: 09/23).

## 1 Introduction

Diabetes mellitus (DM) is an incurable chronic disease characterized by a metabolic syndrome resulting from a lack of insulin and/or its inability to exert its effects. This condition is defined by the presence of hyperglycemia, accompanied by disturbances in the metabolism of carbohydrates, lipids, and proteins [1, 2]; (Bus et al., 2019).

The impact of DM on the global population is significant. In 2021, the International Diabetes Federation (IDF) reported that 537 million adults aged 20 to 79 years were living with DM, representing 10.5% of the population in this age group.

In 2021, Brazil was among the 10 countries with the highest number of adults diagnosed with DM, totaling around 15.7 million in this age group, equivalent to 10.5% of the population. Moreira et al. (2024) conducted a study to predict the prevalence of type 2 diabetes in the Brazilian population using data from the Brazilian Institute of Geography and Statistics (IBGE) and the results indicate a substantial projected increase in the prevalence of the disease among Brazilians aged over 25 years by 2036, reaching 27.0% overall—17.1% in men and 35.9% in women. It is estimated that by 2045, this number will reach 23.2 million [3, 4, 5]. The impact of DM on Brazilian public health is evident, with expenditures directed towards the care of these patients reaching US$42.9 billion in 2021 (BUS et al., 2019).

A concerning aspect of DM is the development of symptomatic peripheral neuropathy in about half of the patients within the first 25 years of the disease. Diabetic neuropathy (DN) and Peripheral Artery Disease (PAD) significantly contribute to the occurrence of foot wounds in diabetic patients [6, 7, 8], (Armstrong et al.2017, Bandyk 2018, Rodrigues et al. 2022). Additionally, body habits, foot structure, and weight distribution also play significant roles in this multifactorial condition (Armstrong et al. 2017; Bandyk 2018; Rodrigues et al. 2022). Another important predictor is the history of previous foot ulcers (Crawford et al. 2015 [9]).

The incidence of foot ulcers in individuals with diabetes can reach 15%, resulting in up to 26 million annual cases and an incidence rate of 2% per year [9]; (Naqvi et al. 2021; Sorber & Abularrage 2021; Rodrigues et al. 2022). These ulcers have a recurrence rate of 65% within 3 years, with 6 in every 1000 patients experiencing annual amputations due to complications [9]; (Sorber & Abularrage 2021; Rodrigues et al. 2022).

More than 50% of ulcers developed by patients with DM can become infected, a problem further exacerbated by complications such as infections from multidrug‐resistant strains, increasing the risk of lower limb amputation, negatively affecting the individual's quality of life, and raising the morbidity and mortality rates in this population [10, 11], (Lucoveis et al., 2018). These resistant strains are particularly responsible for biofilm formation, which is a major factor in the chronicity of wounds, preventing the healing process and prolonging hospital stays, consequently increasing the cost of hospital treatment [4].

In light of these complications, antimicrobial photodynamic therapy (aPDT) emerges as a promising technology in the treatment of chronic wounds. Photodynamic therapy (PDT) involves the application of a photosensitizing agent that, upon activation by a specific light source in the presence of oxygen, generates reactive oxygen species (ROS) capable of eradicating unwanted cells, including tumor, bacterial, viral, and fungal cells [12]. When utilized for the treatment of infections, it is referred to as aPDT [13].

With positive results in combating multidrug‐resistant bacteria and no reported resistance to aPDT in the literature, this approach offers advantages over conventional systemic antimicrobials [14]. Although there are promising studies on the efficacy of aPDT, more clinical research is needed to establish well‐defined protocols [15, 16]; (Carinho et al., 2018) [17, 18].

This study, involving two patients, is driven by the ongoing search for less invasive therapies that have fewer side effects while maintaining the same effectiveness as gold‐standard treatments. The objective of this case report study was to analyze the effect of aPDT using 1% methylene blue as a photosensitizer and a red laser cluster on wound quality and tissue repair in patients with neuropathic diabetic foot ulcers, using the consolidated evaluation scale Bates Jensen Wound Assessment Tool (BWAT).

## 2 Methods

The methodology of the present study was approved by the Research Ethics Committee involving Human Subjects, registered under the numbers: CAAE: 70466823.9.0000.5511 and CAAE: 70466823.9.3001.5279. This report follows the guidelines CARE checklist [19]. Participating patients agreed to sign the informed consent form, which contained comprehensive information regarding their involvement in the study.

The study included patients of both genders presenting with chronic, hard‐to‐heal wounds due to neuropathic diabetic foot complications. These patients had contaminated lesions and BWAT scores between 13 and 60, which corresponds to the operational range considered for this study. The BWAT scale ranges from 13 to 65, with 13 indicating the least severe wound condition and 65 the most severe. Wounds with scores above 60 were excluded, as they are considered extremely severe and typically require referral to specialized care services. The wounds in these patients were primarily the result of amputations necessitated by complications arising from diabetic foot ulcers.

Exclusion criteria comprised patients younger than 18 years, as well as those with wounds originating from causes other than diabetic foot. Patients were also excluded if they had ischemic diabetic foot, defined by an ankle‐brachial index (ABI) below 0.7 or above 1.3, or if they had glycated hemoglobin (HbA1c) levels exceeding 8%.

## 3 Patients Information

### 3.1 Patient 1

A 30‐year‐old male patient with a history of systemic arterial hypertension, type 1 diabetes mellitus diagnosed at age 22, and peripheral neuropathy presented with a puncture wound on the right lower limb (RLL), located between the fourth phalanx and the metatarsal, at a health unit. Following the initial consultation, the patient was prescribed: ceftriaxone 1 g (intramuscular) for 14 days, paracetamol 500 mg (1 tablet every 6 h as needed for pain or fever), ibuprofen 300 mg (2 tablets every 8 h for 5 days), and omeprazole 20 mg (1 tablet for 30 days). About 2 days later, the patient underwent partial amputation of the limb up to the metatarsal region, as recommended by a vascular medical team. Approximately 2 weeks after surgery, the patient developed fever and chills, requiring hospitalization due to worsening infection, which had spread to all phalanges and part of the metatarsal. After discharge, he was referred by the family clinic to begin treatment with aPDT, which started roughly 3 weeks postamputation.

### 3.2 Patient 2

A 56‐year‐old male patient with a diagnosis of type 2 diabetes mellitus, who works as a truck driver and reported wearing rubber boots, sustained a foot injury. He initially did not seek appropriate medical care, leading to a worsening of the wound until he eventually sought emergency treatment. Following evaluation by a vascular specialist, amputation of the second toe was deemed necessary. According to the hospital discharge summary, the patient was admitted with gangrene in the fourth left toe and an abscess on the dorsum of the foot. He underwent an open amputation which included drainage and debridement of the dorsum of the foot, followed by antibiotic therapy. After hospital discharge, the patient sought primary care for wound dressing and was subsequently referred for aPDT treatment, which began approximately 45 days later.

## 4 Initial Clinical Evaluation

### 4.1 Patient 1

**Figure 1:**
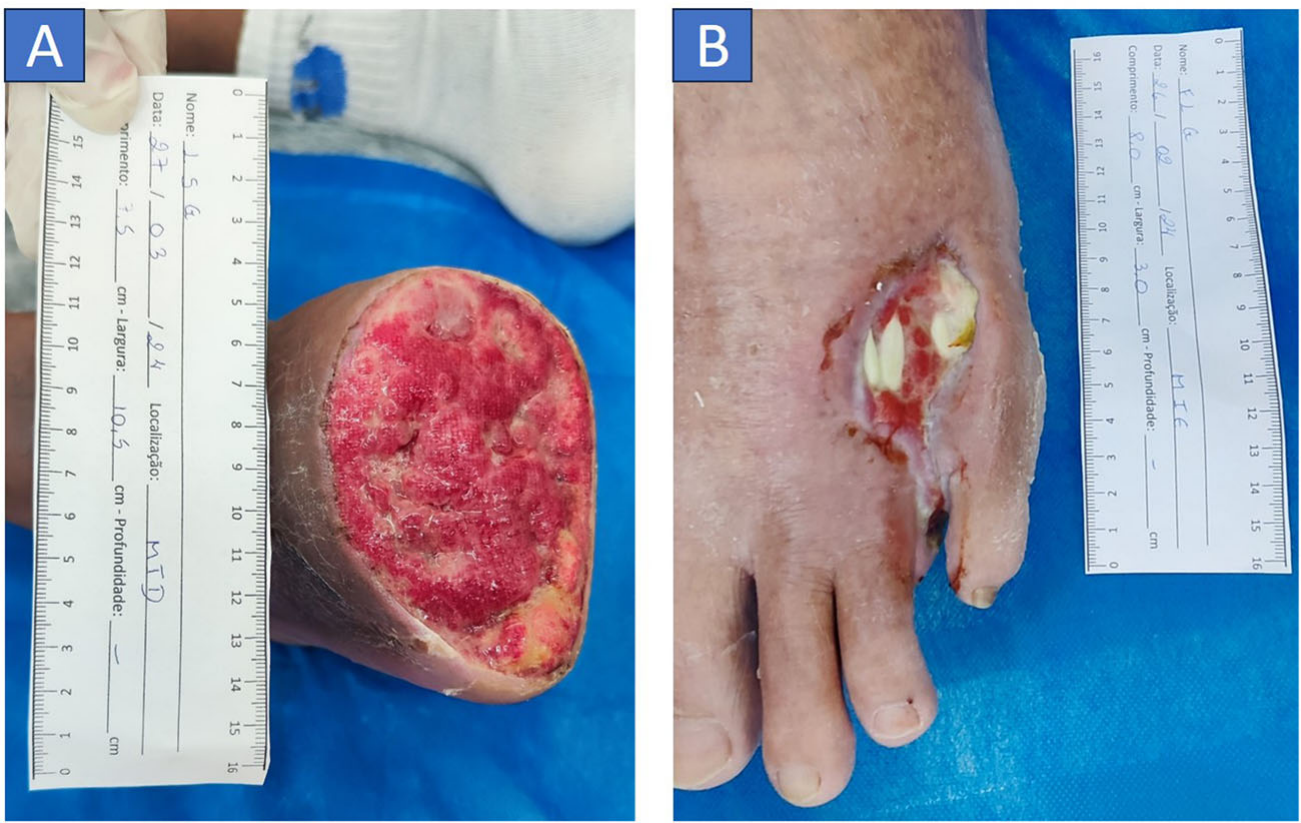
Initial Clinical evaluation of patient 1 (A) and 2 (B).

The patient was alert and oriented, with a glycated hemoglobin level of 7%. He appeared hydrated, afebrile, non‐cyanotic, and non‐icteric, ambulating with difficulty and utilizing crutches due to the amputation. The amputation site exhibited a lesion measuring 53.9 cm², featuring regular edges and a substantial amount of serous and bloody exudate, along with a slight odor. The wound bed comprised 90% granulation tissue and 10% slough, with edema assessed at (++/++++). Edema was assessed using the Godet sign (pitting edema), classified on a scale from 1+ to 4+, based on the depth and duration of the skin depression after pressure is applied. The grades are as follows: 1+ indicates mild edema (≈2 mm depression, immediate rebound), 2+ indicates moderate edema (≈4 mm, short rebound time), 3+ indicates moderately severe edema (≈6 mm, prolonged rebound), and 4+ indicates severe edema (≈8 mm, very delayed rebound) [20]. The perilesional area displayed dryness, scaling, and the presence of ochre dermatitis. The patient reported experiencing intense pain (Figure 1A).

### 4.2 Patient 2

The patient was alert and oriented, with a glycated hemoglobin level of 5.7%. He was hydrated, afebrile, non‐cyanotic, and non‐icteric, ambulating with a limp and using crutches due to an amputation of the left lower limb (LLL). The amputation site exhibited a lesion measuring 11.4 cm², characterized by regular edges and a moderate amount of serous and bloody exudate, along with a slight odor. The wound bed consisted of 90% granulation tissue, 10% slough, and tendon exposure. Edema was noted at (++/++++), with the perilesional area showing dryness and scaling (Figure 1B).

### 4.3 Therapeutic Intervention

**Figure 2:**
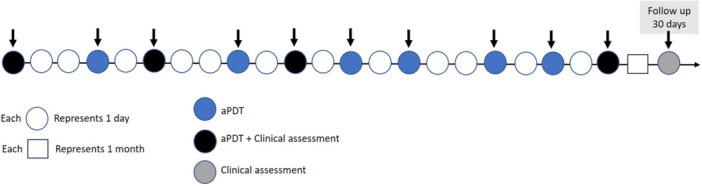
Timeline of intervention and assessment procedures for the case series.

The assessment procedures for the case series are presented in detail on the timeline shown in Figure 2. This figure illustrates the chronological order of interventions, including treatment timings and subsequent evaluations, offering a visual summary of the experimental design and key events throughout the study period.

### 4.4 aPDT Protocol

The wound was initially cleaned with 0.9% saline solution (0.9%) using a 40 x 12 needle and a 500 mL bottle of SF 0.9%. After cleaning, 1% methylene blue aqueous solution [15, 18] was used as a photosensitizer and administered with the aid of a syringe, followed by a pre‐irradiation wait of 5 min.

**Figure 3:**
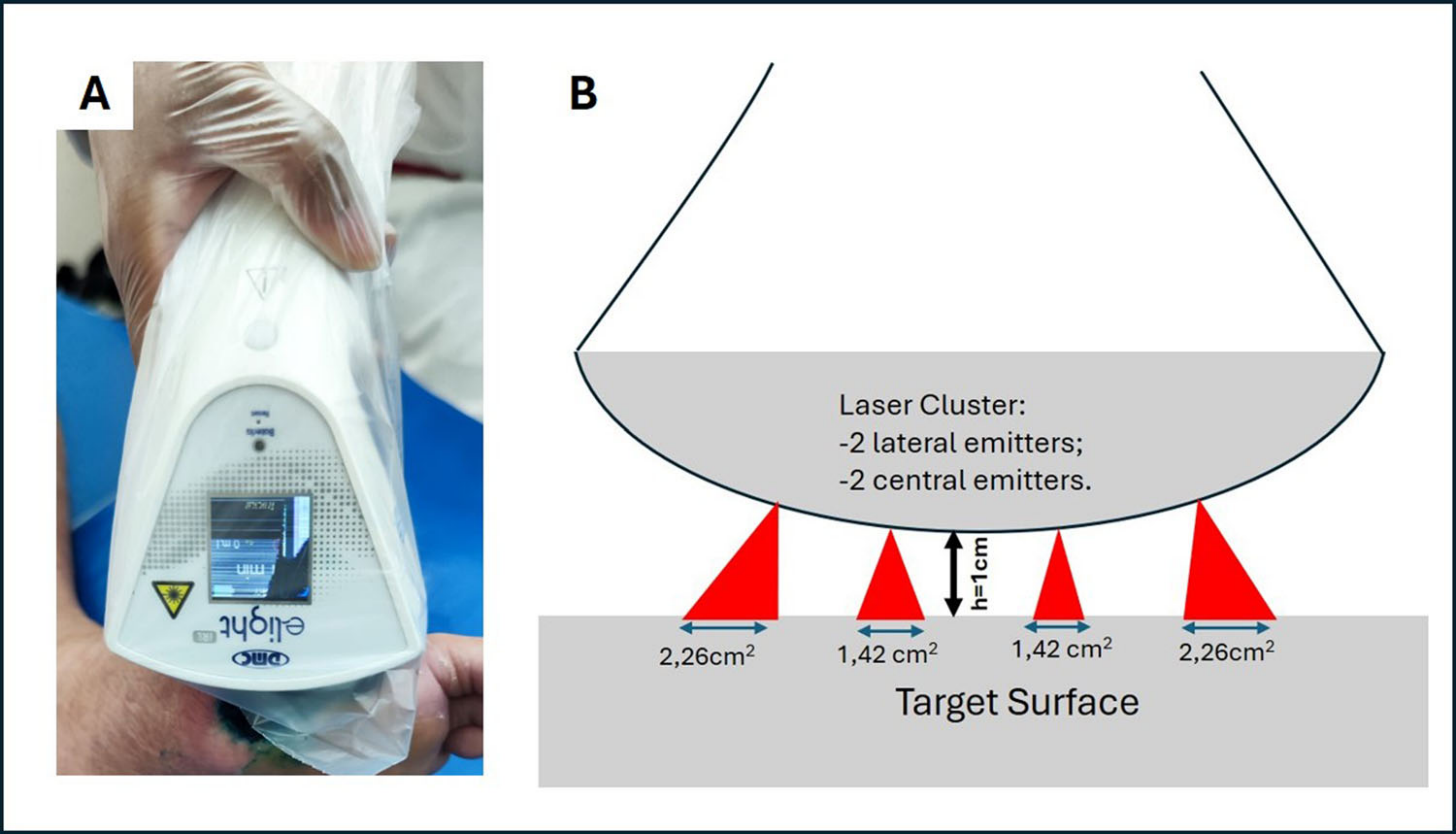
aPDT procedure using the laser cluster device (A) and illustrative image of the equipment highlighting the distinct areas of central and lateral laser emitters (B).

The dosimetric parameters for aPDT were based on a previous study by Ferreira et al. [21], conducted by our research group, which demonstrated positive outcomes in the treatment of diabetic foot ulcers. The protocol included red light irradiation at 660 nm, delivering 6 J per point, administered using the e‐Light IRL cluster device (DMC, São Carlos, SP, Brazil), equipped with four red laser emitters (Figure 3), with a fixed distance of 1 cm between the emitter and the skin, as recommended by the manufacturer. The beam area at the target was 1.42 cm² for central emitters and 2.26 cm² for lateral emitters, resulting in irradiances of 0.070 and 0.044 W/cm², respectively. This corresponds to radiant exposures of 4.2 J/cm² for central emitters and 2.64 J/cm² for lateral emitters over an exposure time of 60 s. All parameters are detailed in Table 1. Following irradiation, a hydrofiber dressing with silver was applied as the primary dressing, and the wound was subsequently covered with sterile gauze, a bandage, and adhesive tape as the secondary dressing.

**Table 1:** Dosimetric parameters used for PDT in the study during each session.

Clusters E‐light IRL – DMC	
Parameter	Value
Peak wavelength (nm)	660
Full spectral width at half height (FWHM) (nm)	2
Operation mode	Continuous
Average radiant power per emitter (mW)	100
Polarization	Linear
Beam area at aperture (cm²) (Exit opening diameter: 1.5 mm)	0.0177
Irradiance at aperture (W/cm²)	5.650
Beam profile	~Gaussian
Application technique	noncontact
Distance (from cluster central point to tissue) (cm)	h = 1
Beam size at target (cm²)	Central emitters: 1,42
	Lateral emitters: 2.26
Irradiance at target (W/cm²)	Central emitters: 0.070
	Lateral emitters: 0.044
Radiant exposure at target (J/cm²)	Central emitters: 4.2
	Lateral emitters: 2.64
Exposure time(s)	60
Number of emitters	4
Radiant energy per emitter (J)	6
Radiant energy per session (J)	24
Number of sessions	10
Frequency of sessions	3x per week (Monday, Wednesday and Friday)
Total radiant energy for the total of sessions (J)	240

A total of 10 aPDT sessions were conducted, three times per week on nonconsecutive days. A follow‐up evaluation was scheduled 30 days after the final session (Figure 2).

### 4.5 Outcome

The primary outcome of this study was the evaluation of wound quality using the BWAT. The BWAT evaluation scale consists of 13 characteristics to assess the wound: size, depth, edges, detachment, type of necrotic tissue, quantity of necrotic tissue, type of exudate, amount of exudate, color of the skin surrounding the wound, edema of the perilesional tissue, hardening of the perilesional tissue, granulation tissue, and epithelialization. Each characteristic is scored from 1 to 5, where a score of 1 indicates optimal wound condition and 5 reflects the worst condition. The total score is calculated by summing the scores of all items, yielding a range of 13 to 65 points, with higher scores signifying more severe wound conditions [22].

Evaluations with the BWAT scale were performed on the first, third, fifth, and tenth sessions of care, and at 30 days following the completion of aPDT treatment, as demonstrated in Figure 2.

The secondary outcomes included:

– The clinical monitoring of the wounds using photographic documentation taken on Sessions 1, 3, 5, 10, and follow up 30 days after. The area of the wound was measured from photographic documentation using the ImageJ (National Institutes of Health‐NIH) software with the hands‐free tool
– The pain intensity was evaluated using a Visual Analog Scale (VAS) to assess changes in pain intensity throughout the treatment process by having patients mark their pain level on a 10 cm line ranging from “no pain” to “worst pain imaginable,” the VAS provided a quantitative measure of subjective pain experiences.

## 5 Results

### 5.1 Quality of the Wound Evaluated by BWAT Scale Tool

The results demonstrated improvement in wound quality for both patients, including a reduction in wound area, edge contraction, diminished odor, decreased slough, reduced secretion, progression of epithelialization, and a considerable reduction in edema. Considering the quality assessed by the BWAT scale (Table 2), both patients experienced an improvement after 10 sessions.

**Table 2:** Patients evaluation of wound quality (Bates‐Jensen Wound Assessment Tool ‐ BWAT), wound area (cm^2^) and Pain Intensity (VAS).

Outcome	Patient	Score Session 1 (S1)	Score Session 3 (S3)	Score Session 5 (S5)	Score Session 10 (S10)	Score follow up 30 days (F30)
Bates‐Jensen Wound Assesment Tool (BWAT)	P1	37	37	34	29	24
	P2	36	34	27	24	14
Wound area (cm^2)^	P1	53.9	44.8	43.9	34.2	13.5
	P2	11.4	9.9	7.4	6.05	0
Pain intensity VAS	P1	10	10	8	5	0
	P2	0	0	0	0	0

For Patient 1 (P1), the score on Session 1 (S1) was 37 and remained unchanged on Session 3 (S3). By Day 5 (D5), it decreased to 34, and by Day 10 (S10), it had further dropped to 29. A follow‐up evaluation after 30 days (F30) confirmed continued progress, with the score reducing to 24.

For Patient 2 (P2), the score on Session 1 (S1) was 36, dropping to 34 on Session 3 (S3). On Session 5 (S5), the score fell to 27, and by Session 10 (S10), it reached 24. At the 30‐day follow‐up (F30), the score had improved to 14, indicating a notable reduction in wound severity over the treatment period.

These findings highlight progressive healing and overall improvement in wound conditions for both patients, as assessed by the Bates‐Jensen Wound Assessment Tool.

Regarding the secondary outcomes, wound area and pain intensity, both parameters showed improvement following aPDT treatment (Table 2). The therapy led to a clear reduction in wound size and pain levels in both patients.

In terms of wound area, both patients exhibited reductions throughout the treatment. Patient 1's wound area decreased from an initial 53.9 cm² on Session 1 to 44.8 cm² by Session 3, 43.9 cm² by Session 5, and 34.2 cm² by Session 10, with a substantial reduction to 13.5 cm² at the 30‐day follow‐up. Similarly, Patient 2 experienced consistent wound healing, with the area shrinking from 11.4 cm² on Session 1 to 9.9 cm² on Session 3, 7.4 cm² on Session 5, and 6.05 cm² on Session 10. By the 30‐day follow‐up, Patient 2's wound had fully closed (0 cm²).

Pain intensity, evaluated by VAS, also declined throughout the treatment. Patient 1 initially reported maximum pain (VAS score of 10) on Session 1 and Session 3, which decreased to 8 on Session 5, 5 on Session 10, and finally resolved to 0 by the 30‐day follow‐up. Patient 2 reported no pain throughout the treatment period, maintaining a consistent VAS score of 0 across all evaluations.

These results confirm that the treatment effectively reduced wound size and pain intensity, with both patients experiencing substantial wound healing and improved comfort.

### 5.2 Clinical Evaluation

The patient 1 presented at the first day with an amputation wound with an area of 34.2 cm², regular edges, minimal serous exudate, characteristic odor, wound bed with 100% granulation tissue, +/+++ edema, and perilesional area with dryness and scaling, with the presence of ochre dermatitis. The patient reported reduced pain compared to the first session (VAS scale 4).

Patient 2 presented at the first day with an amputation wound with an area of 6.05 cm², regular edges, minimal serous exudate, characteristic odor, wound bed with 100% granulation tissue and tendon exposure, no edema, intact perilesional area, and no pain reported.

**Figure 4:**
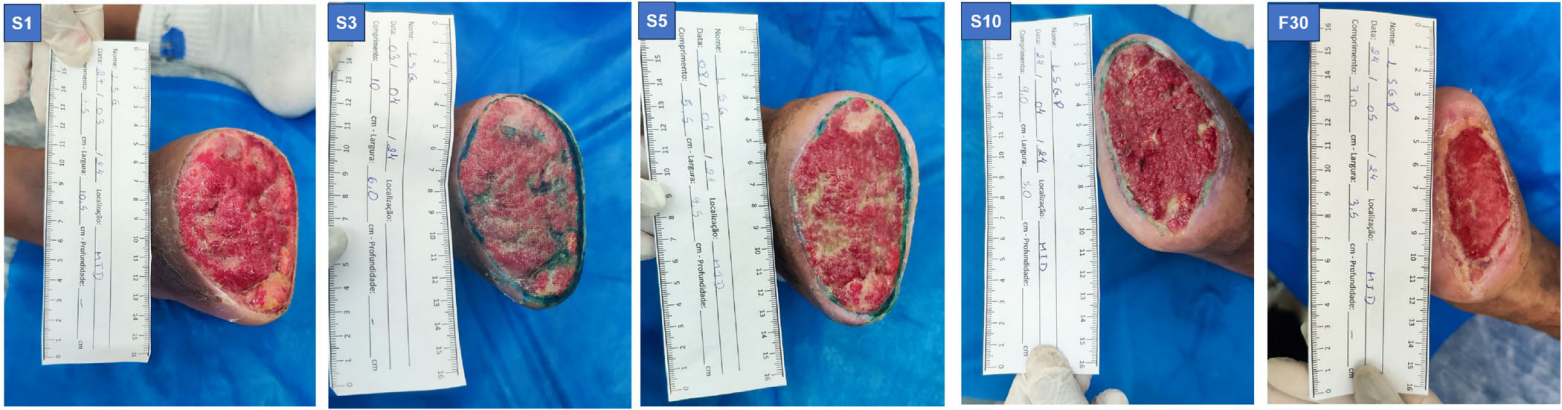
Images of the lesion of Patient 1 at all evaluation time points during the aPDT procedure.

**Figure 5:**
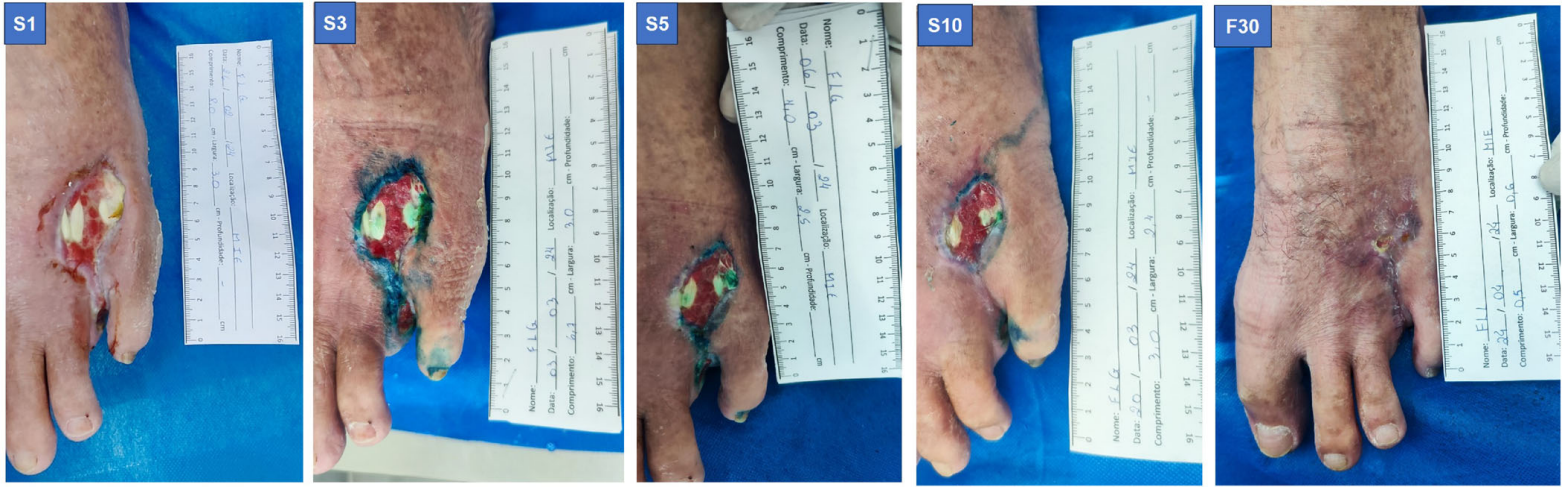
Images of the lesion in Patient 2 at all evaluation time points during the aPDT procedure.

After treatment, the results showed an improvement in wound quality for both patients, including reduced wound area, edge contraction, diminished odor, decreased slough, reduced exudate, advanced epithelialization, and a marked reduction in edema (Figures 4 and 5).

Furthermore, the patients did not experience any complications during the treatment, adhering to the proposed protocol.

### 5.3 Follow‐Up After 30 Days

A new evaluation was performed using the BWAT scale in a 30‐day follow‐up, showing a reduction in the score (Table 2). The wound quality remained consistent. After 30 days from the last session, both patients reported no pain (Table 2; Figures 4 and 5).

## 6 Discussion

Current standard treatments, while effective, can be invasive and associated with various side effects. Therefore, exploring alternative treatments like aPDT offers the potential for a less invasive approach that could improve patient outcomes, reduce the burden on healthcare systems, and provide a comparable or superior therapeutic effect. This study contributes to the growing body of evidence supporting aPDT as a viable, effective, and well‐tolerated option for the management of chronic wounds, particularly in patients with diabetic foot ulcers. In addition to being a painless therapy with no reported adverse effects, aPDT demonstrated positive results in wound progression, even in a complex case involving prior amputation. These findings highlight the potential of aPDT as an adjunctive therapy in primary care settings, offering a safe and accessible approach to enhance healing in patients with advanced complications.

The study highlights the importance of wound monitoring and correct use of dosimetry parameters, optimizing treatment outcomes with aPDT, using a wavelength of 660 nm (red), an average radiant power of 100 mW, and 6 J of radiant energy per emitter. For this study, a cluster device E‐light IRL (DMC, São Carlos, SP, Brazil) was used, adhering to recent national manufacturing standards. The use of cluster devices, as in this study, appears promising by allowing the treatment of extensive wound areas, optimizing professional time, and offering effective results.

Methylene blue strongly absorbs red light, has good production of ROS, low toxicity, low cost, and is readily available in the market [23, 24, 25]. The use of aPDT has shown promising results in recent studies. This case report presents aPDT as an adjunctive safety therapy [14, 26], demonstrating excellent responses in patients with DM. Unlike other studies, the novelty of this case report lies in its focus on patients who had already undergone lower limb amputations associated with diabetes, an area that has not been extensively explored. Additionally, our study incorporates a clinical temporal evaluation to assess the progression of wound healing over time, offering insights into the long‐term efficacy of aPDT in patients with advanced complications.

The patients in this study did not require antibiotics during the treatment; only primary care follow‐up and traditional wound care in conjunction with aPDT were sufficient for positive outcomes within a short timeframe. These patients were specifically selected for the study due to their advanced condition—both had lower limb amputations related to diabetes. This clinical focus adds a unique perspective to the use of aPDT in complex cases.

Some studies have highlighted the promising potential of aPDT in the treatment of diabetic ulcers using methylene blue. Tardivo et al. [15] conducted a clinical trial involving patients with Grade 3 ulcers and osteomyelitis to evaluate the efficacy of incorporating aPDT into conventional antibiotic treatment. aPDT was administered using a 1:1 mixture of methylene blue and 1% toluidine blue in water, utilizing light sources including LEDs (640 nm, 50 mW/cm², 30 J/cm²) for external application and fiber optic white light (6 J/cm², 10 mW/cm²) for internal application. This combined treatment significantly reduced the necessity for amputations, with 17 out of 18 patients in the experimental group achieving complete recovery, whereas all patients in the control group required amputation. Ferreira et al. [21] treated diabetic foot ulcers in 21 patients using aPDT with 0.01% methylene blue and laser irradiation (660 nm, 100 mW, 6 J per point) over 4 to 13 sessions. They observed an average reduction of 5.8 points in the overall score on the BWAT scale and a decrease in the lesion area between pre‐ and posttreatment assessments. In the present study, Patient 1 showed a 13‐point improvement, indicating wound progress, while Patient 2 demonstrated a 22‐point improvement, achieving complete healing.

Pain reduction was another notable result, evidenced by the substantial drop in VAS scores for Patient 1 and the absence of pain in Patient 2 throughout treatment. Pain often acts as a barrier to wound healing and impacts patients’ quality of life; therefore, the decrease in pain intensity during treatment with the aPDT protocol is clinically relevant. In a meta‐analysis that evaluated pain using the VAS scale in 458 people with foot ulcers related to diabetes who underwent PDT, it concluded that no differences were observed after treatment. The results of this study highlight the potential effectiveness of aPDT in managing wound healing, consequently reducing pain. However, it is not possible to state that there is a relationship between pain reduction and the application of aPDT [27].

Both patients exhibited reductions (of 74.9% P1% and 100% P2) in wound area, with one patient achieving complete closure at 30‐day follow‐up. The progressive decrease in wound area suggests that aPDT may support accelerated healing, particularly in complex or hard‐to‐heal wounds. This improvement in wound area aligns with previous research indicating that aPDT's antimicrobial and anti‐inflammatory effects create a favorable environment for tissue repair and wound contraction [15, 16]; (Carinho et al., 2018) [17, 18].

Carrinho et al. [28] found that aPDT with 0.01% MB (60 nm, 30 mW, 6 J/cm^2^, 6 s) when combined with conventional treatment led to a greater reduction in wound size compared to conventional treatment alone, indicating enhanced tissue repair. Ferreira et al. [21], already mentioned, also observed a decrease (from an average of 12.8 to an average of 8.18) in the lesion area between pre‐ and posttreatment assessments.

Like the current study, none of the previous studies reported adverse effects associated with the use of aPDT in the treatment of diabetic foot wounds. Foot lesions in individuals with DM represent a public health issue [29]. The International Diabetes Federation (IDF) projects that the global adult population affected by DM could reach 643 million by 2030 and 783 million by 2045 [4]. Beyond the public and individual costs, the emotional impacts of amputation and the inability to perform daily and work activities are also noteworthy. Given these promising results, further clinical studies with larger sample sizes are essential to establish concrete evidence for aPDT's efficacy and safety. These studies would provide data to support standardized protocols for wound care, ultimately guiding healthcare professionals in incorporating aPDT into routine practice with confidence in its scientific evidence.

In conclusion, aPDT was effective across all evaluated outcomes in this study. The treatment improved wound quality, as evidenced by reduced BWAT scores, a 74.9% reduction in wound area in patient 1, and complete wound healing in patient 2. These findings suggest that aPDT may be a valuable therapeutic option for complex diabetic foot wounds and underscore the need for further research to confirm its efficacy and safety in broader clinical settings. Given the increasing demand for effective treatments for diabetic foot ulcers, advancing research on light‐based technologies as therapeutic tools in wound care is essential. These approaches may improve clinical outcomes while offering safer and more supportive options for healthcare professionals utilizing complementary therapies.

## Ethics Statement

The project received approval from the Research Ethics of Universidade Nove de Julho (process: 70466823.9.0000.5511).

## Consent

All the participants included signed an Informed consent. The CI document was in writing.

## Conflicts of Interest

The authors declare no conflicts of interest.

## Data Availability

All data will be available for the readers.

## References

[1] B. A. Lipsky , É. Senneville , Z. G. Abbas , et al., International Working Group on the Diabetic Foot (IWGDF) ., “Guidelines on the Diagnosis and Treatment of Foot Infection In Persons With Diabetes (IWGDF 2019 Update),” Diabetes/Metabolism Research and Reviews 36, no. Suppl 1 (March 2020): e3280, 10.1002/dmrr.3280. 32176444

[2] A. J. M. Boulton , “The Diabetic Foot: Grand Overview, Epidemiology and Pathogenesis,” Diabetes/Metabolism Research and Reviews 24, no. Suppl 1 (May/June 2008): S3–S6, 10.1002/dmrr.833. 18442166

[3] J. A. C. Lira , L. T. Nogueira , B. M. A. Oliveira , D. D. R. Soares , A. M. R. D. Santos , and T. M. E. Araújo , “Factors Associated With the Risk of Diabetic Foot in Patients With Diabetes Mellitus in Primary Care,” Revista da Escola de Enfermagem da U S P 55 (2021 July 26): e03757. Portuguese, English, 10.1590/S1980-220X2020019503757. 34320142

[4] D. J. Magliano and E. J. Boyko , IDF Diabetes Atlas 10th edition Scientific Committee ., IDF Diabetes Atlas [Internet] (International Diabetes Federation, 2021). 10th ed. 35914061

[5] J. Muzy , M. R. Campos , I. Emmerick , R. S. D. Silva , and J. M. A. Schramm , “Prevalence of Diabetes Mellitus and Its Complications and Characterization of Healthcare Gaps Based on Triangulation of Studies,” Cadernos de saúde pública/Ministério da Saúde, Fundação Oswaldo Cruz, Escola Nacional de Saúde Pública 37, no. 5 (May 2021): e00076120, 10.1590/0102-311X00076120. 34076095

[6] D. Selvarajah , D. Kar , K. Khunti , et al., “Diabetic Peripheral Neuropathy: Advances in Diagnosis and Strategies for Screening and Early Intervention,” Lancet Diabetes & Endocrinology 7, no. 12 (December 2019): 938–948, 10.1016/S2213-8587(19)30081-6. 31624024

[7] M. Volmer‐Thole and R. Lobmann , “Neuropathy and Diabetic Foot Syndrome,” International Journal of Molecular Sciences 17, no. 6 (June 2016): 917, 10.3390/ijms17060917. 27294922 PMC4926450

[8] A. J. Boulton , “Diabetic Neuropathy and Foot Complications,” Handb Clin Neurol 126 (2014): 97–107, 10.1016/B978-0-444-53480-4.00008-4. 25410217

[9] S. A. Bus , J. J. Van Netten , R. J. Hinchliffe , J. Apelqvist , B. A. Lipsky , and N. C. Schaper , “IWGDF Editorial Board. Standards for the Development and Methodology of the 2019 International Working Group on the Diabetic Foot Guidelines,” Diabetes/Metabolism Research and Reviews 36, no. Suppl 1 (March 2020): e3267, 10.1002/dmrr.3267. 31916377

[10] M. Monteiro‐Soares , E. J. Boyko , J. Ribeiro , I. Ribeiro , and M. Dinis‐Ribeiro , “Risk Stratification Systems for Diabetic Foot Ulcers: A Systematic Review,” Diabetologia 54, no. 5 (May 2011): 1190–1199, 10.1007/s00125-010-2030-3. 21249490

[11] A. J. M. Boulton , “The Pathway to Foot Ulceration in Diabetes,” Medical Clinics of North America 97, no. 5 (September 2013): 775–790, 10.1016/j.mcna.2013.03.007. 23992891

[12] M. R. Hamblin and T. Hasan , “Photodynamic Therapy: A New Antimicrobial Approach to Infectious Disease?,” Photochemical & Photobiological Sciences 3, no. 5 (May 2004): 436–450, 10.1039/b311900a. 15122361 PMC3071049

[13] V. Nesi‐Reis , D. S. S. L. Lera‐Nonose , J. Oyama , et al., “Contribution of Photodynamic Therapy in Wound Healing: A Systematic Review,” Photodiagnosis and Photodynamic Therapy 21 (March 2018): 294–305, 10.1016/j.pdpdt.2017.12.015. 29289704

[14] A. Warrier , N. Mazumder , S. Prabhu , K. Satyamoorthy , and T. S. Murali , “Photodynamic Therapy to Control Microbial Biofilms,” Photodiagnosis and Photodynamic Therapy 33 (March 2021): 102090, 10.1016/j.pdpdt.2020.102090. 33157331

[15] J. P. Tardivo , F. Adami , J. A. Correa , M. A. S. Pinhal , and M. S. Baptista , “A Clinical Trial Testing the Efficacy of PDT in Preventing Amputation in Diabetic Patients,” Photodiagnosis and Photodynamic Therapy 11, no. 3 (September 2014): 342–350, 10.1016/j.pdpdt.2014.04.007. 24814697

[16] L. P. Rosa , F. C. da Silva , R. L. Vieira , et al., “Application of Photodynamic Therapy, Laser Therapy, and a Cellulose Membrane for Calcaneal Pressure Ulcer Treatment in a Diabetic Patient: A Case Report,” Photodiagnosis and Photodynamic Therapy 19 (Septemeber 2017): 235–238, 10.1016/j.pdpdt.2017.06.011. 28666974

[17] X. Li , H. Kou , C. Zhao , F. Zhu , Y. Yang , and Y. Lu , “Efficacy and Safety of ALA‐PDT in Treatment of Diabetic Foot Ulcer With Infection,” Photodiagnosis and Photodynamic Therapy 38 (June 2022): 102822, 10.1016/j.pdpdt.2022.102822. 35331957

[18] G. B. Cesar , A. P. Winyk , F. Sluchensci Dos Santos , et al., “Treatment of Chronic Wounds With Methylene Blue Photodynamic Therapy: A Case Report,” Photodiagnosis and Photodynamic Therapy 39 (September 2022): 103016, 10.1016/j.pdpdt.2022.103016. 35840009

[19] CARE Statement . (n.d.). The CARE Guidelines: A Checklist for Case Reports. Retrieved September 30, 2024, https://www.care-statement.org/.

[20] N. C. M. Silva , É. C. L. Chaves , E. C. Carvalho , and D. H. Iunes , “Instrumento Para Avaliação Da Integridade Tissular Dos Pés De Portadores De Diabetes Melittus,” Acta Paulista de Enfermagem 26, no. 6 Dez (2013): 535–541, 10.1590/S0103-21002013000600005.

[21] R. C. Ferreira , R. B. Cecatto , S. T. Perez , et al., “Adjuvant Effect of Antimicrobial Photodynamic Therapy (aPDT) in the Treatment of Diabetic Foot Ulcers: A Case Series,” Journal of Biophotonics 17, no. 4 (April 2024): e202300412, 10.1002/jbio.202300412. 38253349

[22] D. F. S. Alves , A. O. Almeida , J. L. G. Silva , F. I. Morais , S. R. P. E. Dantas , and N. M. C. Alexandre , “Translation and Adaptation of The Bates‐Jensen Wound Assessment Tool for The Brazilian Culture,” Texto & Contexto ‐ Enfermagem 24, no. 3 (July 2015): 826–833, 10.1590/0104-07072015001990014.

[23] R. Boltes Cecatto , L. Siqueira de Magalhães , M. Fernanda Setúbal Destro Rodrigues , et al., “Methylene Blue Mediated Antimicrobial Photodynamic Therapy in Clinical Human Studies: The State of the Art,” Photodiagnosis and Photodynamic Therapy 31 (September 2020): 101828, 10.1016/j.pdpdt.2020.101828. 32473398

[24] J. P. Tardivo and M. S. Baptista , “Treatment of Osteomyelitis in the Feet of Diabetic Patients by Photodynamic Antimicrobial Chemotherapy,” Photomedicine and Laser Surgery 27, no. 1 (February 2009): 145–150, 10.1089/pho.2008.2252. 19196112

[25] J. P. Tardivo , A. Del Giglio , C. S. de Oliveira , et al., “Methylene Blue In Photodynamic Therapy: From Basic Mechanisms to Clinical Applications,” Photodiagnosis and Photodynamic Therapy 2, no. 3 (September 2005): 175–191, 10.1016/S1572-1000(05)00097-9. 25048768

[26] X. Shen , L. Dong , X. He , et al., “Treatment of Infected Wounds With Methylene Blue Photodynamic Therapy: An Effective and Safe Treatment Method,” Photodiagnosis and Photodynamic Therapy 32 (December 2020): 102051, 10.1016/j.pdpdt.2020.102051. 33059110

[27] C. Hou , L. Zhang , L. Wang , et al., “A Meta‐Analysis and Systematic Review of Photodynamic Therapy for Diabetic Foot Ulcers,” Photodiagnosis and Photodynamic Therapy 48 (August 2024): 104228, 10.1016/j.pdpdt.2024.104228. 38866070

[28] P. M. Carrinho , D. I. K. Andreani , V. A. Morete , S. Iseri , R. S. Navarro , and A. B. Villaverde , “A Study on the Macroscopic Morphometry of the Lesion Area on Diabetic Ulcers in Humans Treated With Photodynamic Therapy Using Two Methods of Measurement,” Photomedicine and Laser Surgery 36, no. 1 (January 2018): 44–50, 10.1089/pho.2017.4305. 29023192

[29] J. Muzy , M. R. Campos , I. Emmerick , and R. Sabino , “Oferta E Demanda De Procedimentos Atribuíveis Ao Diabetes Mellitus E Suas Complicações no Brasil,” Ciência & saúde coletiva 27, no. 4 (April 2022): 1653–1667. Portuguese, English, 10.1590/1413-81232022274.05612021. 35475843

